# Endovascular thrombectomy in patients with acute ischemic stroke and prestroke mRS 3: a multicenter IPTW analysis focused on functional preservation

**DOI:** 10.3389/fneur.2026.1841144

**Published:** 2026-05-22

**Authors:** Sangwon Choi, Minwoo Lee, Mi Sun Oh, Yerim Kim, Chulho Kim, Hee-Jin Im, Sang-Hwa Lee

**Affiliations:** 1 Department of Neurology, Hallym University Sacred Heart Hospital, Hallym University College of Medicine, Anyang, Republic of Korea; 2 Department of Neurology, Kangdong Sacred Heart Hospital, Hallym University College of Medicine, Seoul, Republic of Korea; 3 Department of Neurology, Chuncheon Sacred Heart Hospital, Hallym University College of Medicine, Chuncheon, Republic of Korea; 4 Department of Neurology, Dongtan Sacred Heart Hospital, Hallym University College of Medicine, Hwaseong, Republic of Korea

**Keywords:** acute ischemic stroke, endovascular treatment (EVT), intravenous thrombolysis (IVT), inverse probability of treatment assignment weighting (IPTW), large vessel occlusion, prestroke disability

## Abstract

**Background:**

Evidence for reperfusion therapy in patients with acute ischemic stroke and prestroke disability remains limited because individuals with prestroke modified Rankin Scale (mRS) score of 3 have largely been excluded from randomized trials. We evaluated the effectiveness and safety of reperfusion strategies in this population.

**Methods:**

We conducted a multicenter registry-based study including patients with acute ischemic stroke due to large vessel occlusion (LVO) and prestroke mRS score of 3 who presented within 4.5 h of symptom onset. Patients were categorized into medical treatment (MT), intravenous thrombolysis (IVT), or endovascular thrombectomy (EVT). The primary outcome was return to prestroke functional status at 3 months (mRS = 3). Secondary outcomes included ordinal mRS shift, symptomatic hemorrhagic transformation (sHT), and mortality. To reduce treatment-selection bias, inverse probability of treatment weighting (IPTW) based on multinomial propensity scores was applied.

**Results:**

Among 183 patients, 101 received MT, 37 IVT, and 45 EVT. After IPTW adjustment, EVT was associated with a significantly higher likelihood of returning to prestroke functional status compared with MT (OR 7.63, 95% CI 3.02–19.28), whereas IVT showed no significant benefit (OR 1.15, 95% CI 0.34–3.85). EVT was also associated with a favorable shift across the full mRS distribution (common OR 0.32, 95% CI 0.14–0.70). Neither EVT nor IVT increased the risk of sHT or mortality.

**Conclusions:**

EVT was associated with substantial functional benefit without increased safety risk in patients with LVO and prestroke mRS 3, supporting consideration of EVT in selected patients with moderate prestroke disability.

## Introduction

Although reperfusion therapy is well established for patients with acute ischemic stroke due to large vessel occlusion (LVO) and pre-stroke modified Rankin Scale (mRS) scores of 0–2, evidence remains limited for patients with pre-existing disability. Patients with pre-stroke mRS scores of 3 have largely been excluded from major randomized trials (1–7). Nevertheless, registry-based studies suggest that patients with moderate pre-stroke disability constitute a significant proportion of acute ischemic stroke cases in real-world practice. According to observational registries, around 10%−20% of acute ischemic stroke patients with LVO have moderate pre-stroke disability (mRS≥3) at presentation (8). As populations continue to age, this subgroup is expected to grow, highlighting the increasing clinical importance of treatment strategies that aim to preserve baseline functional status rather than achieve full neurological recovery (9–11).

Accumulating observational evidence suggests that reperfusion therapies may offer functional advantages to certain patients with pre-stroke disability, especially those who undergo endovascular treatment (EVT) (12–16). However, the existing literature is limited by heterogeneous inclusion criteria, variable definitions of functional outcomes, and substantial confounding by indication. Patients selected for EVT often have more severe strokes, making direct comparisons with medically treated patients susceptible to bias. Furthermore, few studies have simultaneously evaluated medical treatment, intravenous thrombolysis (IVT), and EVT within a single cohort of patients with a pre-stroke mRS score of 3 and confirmed LVO, which limits the interpretability and generalizability of the available data.

To address these gaps, we conducted a multicenter, registry-based study to evaluate the effectiveness and safety of reperfusion therapies in patients with acute ischemic stroke due to LVO and a pre-stroke mRS score of 3.

To reduce treatment selection bias, an issue inherent to observational research, we applied inverse probability of treatment weighting (IPTW) based on multinomial propensity scores. This approach allowed us to make balanced comparisons among medical treatment, IVT, and EVT. We hypothesized that EVT would be associated with a higher likelihood of returning to pre-stroke functional status without increasing the risk of symptomatic hemorrhagic transformation or mortality.

## Methods

### Study population

This was a retrospective observational study using prospectively collected data from a multicenter stroke registry involving four tertiary stroke centers in South Korea. The registry consecutively enrolled patients with acute ischemic stroke (AIS) using a standardized data-collection protocol that captured demographic information, vascular risk factors, acute treatment details, neuroimaging findings, and functional outcomes at 3 months. The study period spanned from September 2015 to March 2025. Patients were eligible for inclusion if they met all of the following criteria: (1) age ≥18 years; (2) prestroke mRS score of 3; (3) arrival at the hospital within 4.5 h from last known well; and (4) AIS caused by LVO confirmed by vascular imaging. LVO was defined as occlusion of the intracranial internal carotid artery, middle cerebral artery (M1 or proximal M2), vertebral artery, basilar artery, or posterior cerebral artery.

**Figure 1:**
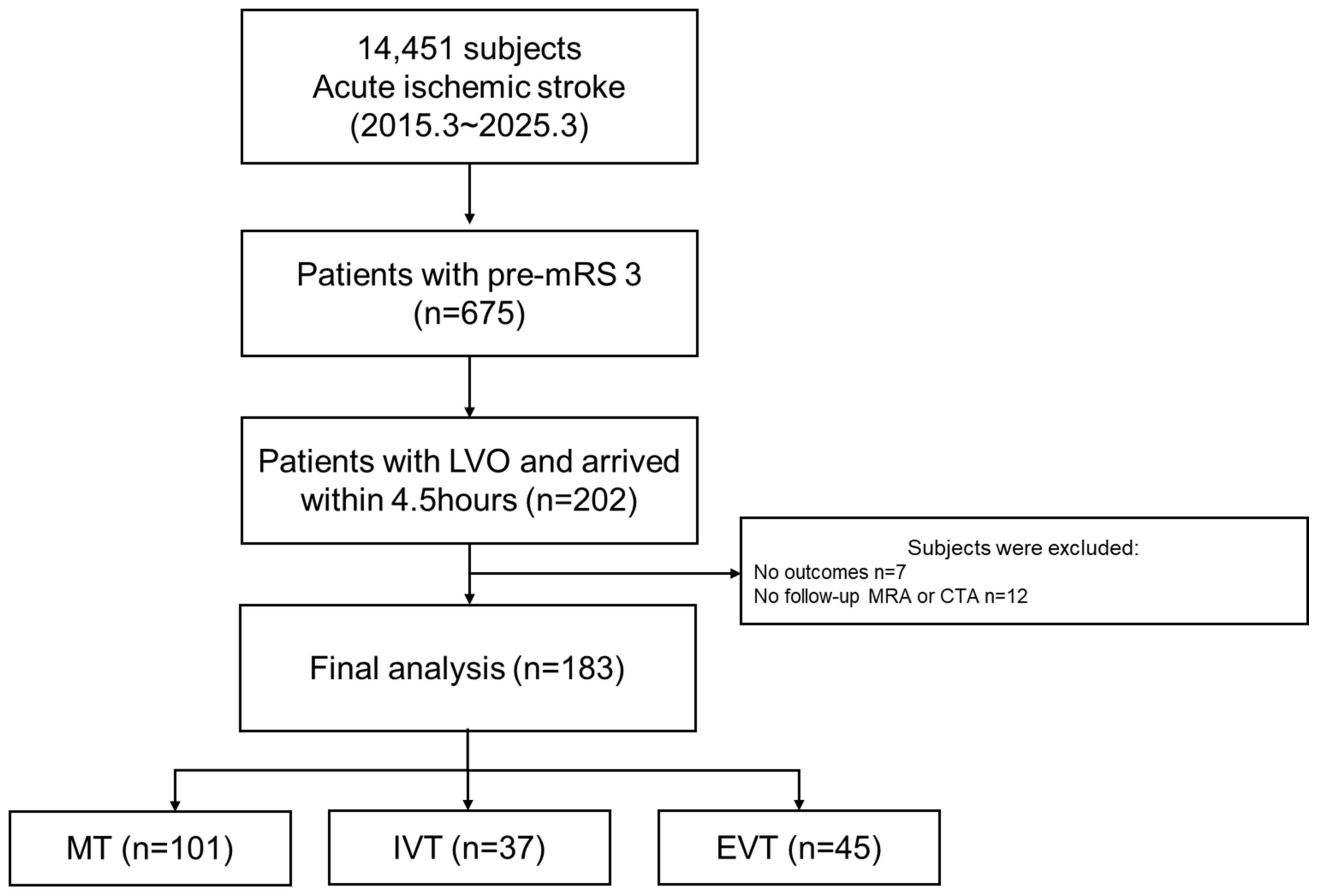
Study flow chart. mRS, modified Rankin Scale; LVO, large vessel occlusion; MRA, magnetic resonance angiograph; CTA, computed tomography angiography; MT, medical treatment; IVT, intravenous thrombolysis; EVT, endovascular thrombectomy.

Of the 14,451 patients with AIS who were screened during the study period, 675 had a pre-stroke mRS score of 3. Of these patients, approximately 30% (202/675) had confirmed LVO and arrived within the therapeutic time window. Ultimately, 183 patients were included in the final analytic cohort (Figure 1).

### Definition of parameters

The primary exposure of interest was the acute treatment strategy, which was categorized into three mutually exclusive groups: (1) medical treatment alone (MT); (2) IVT without EVT; and (3) EVT, including EVT alone or EVT combined with bridging IVT. Attending stroke physicians made treatment decisions according to contemporaneous clinical guidelines and institutional protocols. Demographic characteristics, clinical variables, laboratory findings, and stroke outcomes were extracted directly from a web-based registry that was maintained prospectively. Initial stroke severity was assessed by trained neurologists using the National Institutes of Health Stroke Scale (NIHSS). Stroke etiology was determined using a modified Trial of ORG 10,172 in Acute Stroke Treatment (TOAST) classification system (17). Two expert vascular neurologists (C. Kim and S.-H. Lee) identified successful reperfusion and Alberta Stroke Program Early CT Score (ASPECTS). Successful reperfusion was defined as a TICI grade of 2b or 3 for patients undergoing EVT. This was confirmed by follow-up magnetic resonance angiography (MRA) or computed tomography angiography (CTA), performed at least 48 h after presentation, for patients undergoing IVT or MT (interclass correlation coefficient [ICC], 0.92; *p* < 0.001).

### Outcome measures

The primary outcome was good functional outcome at 3 months, defined as return to prestroke functional status (3-month mRS score of 3). This outcome was chosen because the primary therapeutic goal for patients with moderate pre-stroke disability is to preserve baseline functional independence rather than to achieve full neurological recovery.

The secondary outcomes were symptomatic hemorrhagic transformation (sHT) during hospitalization and all-cause mortality at 3 months. sHT was defined as radiologically confirmed hemorrhagic transformation on follow-up computed tomography (CT) or magnetic resonance imaging (MRI) accompanied by neurological deterioration within 3 days of hospitalization, according to the European Cooperative Acute Stroke Study criteria. Two blinded vascular neurologists (C. Kim and S.-H. Lee) independently adjudicated sHT events (ICC, 0.88; *p* < 0.001).

### Statistical analysis

Baseline characteristics were summarized using medians and interquartile ranges for continuous variables, as well as frequencies and percentages for categorical variables. Group comparisons were performed using Kruskal–Wallis tests for continuous variables and chi-square or Fisher's exact tests for categorical variables, as appropriate.

To mitigate the confounding effects of indication inherent to observational studies, we estimated multinomial propensity scores using the following variables: age, sex, admission NIHSS score, onset-to-arrival time, ASPECTS, and TOAST classification. We applied IPTW with stabilized weights to estimate average treatment effects across the three treatment groups (MT, IVT, and EVT). We assessed covariate balance before and after weighting using standardized mean differences, with values < 0.1 indicating adequate balance.

Primary and secondary outcomes were analyzed using IPTW-weighted logistic regression models with robust variance estimation. We conducted ordinal mRS shift analysis using IPTW-weighted proportional odds models to assess distributional shifts in functional outcomes across the full range of mRS scores.

As sensitivity analyses, conventional multivariable logistic regression models were fitted using the same covariates. For outcomes with sparse events or evidence of separation, additional Firth penalized logistic regression was performed to obtain bias-reduced estimates. All statistical tests were two-sided, and a *p*-value less than 0.05 was considered statistically significant. The statistical analyses were conducted using R and Python software. The study adhered to the STROBE guidelines and the Declaration of Helsinki.

## Results

### Baseline characteristics

Initially, 202 patients met the study inclusion criteria. However, 19 patients were excluded due to missing outcome or follow-up imaging data. There was no significant difference in the distribution of these excluded patients across the initially allocated treatment groups (MT: 9/110 [8.2%]; IVT: 6/43 [14.0%]; EVT: 4/49 [8.2%]; *p* = 0.52), suggesting that this exclusion was unlikely to introduce substantial selection bias. Consequently, a total of 183 patients with acute ischemic stroke due to large vessel occlusion and a prestroke modified Rankin Scale (mRS) score of 3 were included in the analysis, of whom 101 received MT alone, 37 received IVT, and 45 underwent EVT (Figure 1). Baseline characteristics are summarized in Table 1. There were no significant differences in mean age, sex distribution, onset-to-arrival time, or ASPECTS among the three groups. However, stroke severity differed substantially by treatment strategy. Patients treated with EVT or IVT presented with significantly higher initial NIHSS scores than those receiving medical treatment (median NIHSS: 16.0 for EVT, 15.0 for IVT, and 8.0 for MT; *p* < 0.001). Atrial fibrillation was markedly more prevalent in the IVT group (81.1%) than in the MT (27.3%) and EVT (22.2%) groups (*p* < 0.001). Current smoking was observed exclusively in the MT and EVT groups (*p* = 0.035). Stroke mechanism and lesion location were similar across treatment groups. Successful reperfusion was significantly more common in patients receiving IVT (45.9%) or EVT (60.0%) than in those receiving MT alone (7.9%; *p* < 0.001).

**Table 1:** Baseline characteristics according to treatment groups.

Variables	MT (*n* = 101)	IVT (*n* = 37)	EVT (*n* = 45)	*p*-value
Age, years, mean (SD)	77.4 (12.2)	80.6 (12.4)	81.1 (11.3)	0.15
Male sex, *n* (%)	44 (43.6%)	18 (48.6%)	21 (46.7%)	0.15
Onset-to-arrival time, h [IQR]	1.3 [0.8–2.7]	1.0 [0.9–1.8]	1.8 [0.9–4.3]	0.21
ASPECTS, score [IQR]	7.0 [7.0–8.0]	8.0 [7.0–8.0]	8.0 [7.0–8.0]	0.99
Initial NIHSS, score [IQR]	8.0 [3.0–14.0]	15.0 [10.0–20.0]	16.0 [12.0–17.0]	<0.001
History of stroke, *n* (%)	47 (46.5)	10 (27.0)	21 (46.7)	0.10
History of CHD, *n* (%)	13 (12.9)	9 (24.3)	7 (15.6)	0.26
Hypertension, *n* (%)	86 (85.1)	34 (91.9)	35 (77.8)	0.21
Diabetes mellitus, *n* (%)	43 (42.6)	13 (35.1)	17 (37.8)	0.69
Atrial fibrillation, *n* (%)	27 (27.3)	30 (81.1)	10 (22.2)	<0.001
Hyperlipidemia, *n* (%)	27 (26.7)	9 (24.3)	15 (33.3)	0.62
Current smoking, *n* (%)	16 (15.8)	0 (0.0)	5 (11.1)	0.035
Prior antiplatelet use, *n* (%)	35 (34.7)	13 (35.1)	23 (51.1)	0.15
Prior anticoagulation use, *n* (%)	14 (13.9)	8 (21.6)	4 (8.9)	0.26
Prior statin use, *n* (%)	37 (36.6)	12 (32.4)	22 (48.9)	0.25
Stroke mechanism, *n* (%)				
LAA	50 (49.5)	12 (32.4)	26 (57.8)	0.095
CE	29 (28.7)	18 (48.6)	10 (22.2)	
Others	22 (21.8)	7 (18.9)	9 (20.0)	
Lesion location, *n* (%)				
Anterior circulation	81 (80.2)	27 (73.0)	36 (80.0)	0.70
Posterior circulation	15 (14.9)	6 (16.2)	5 (11.1)	
Multiple	5 (5.0)	4 (10.8)	4 (8.9)	
Successful reperfusion, *n* (%)	8 (7.9)	17 (45.9)	27 (60.0)	<0.001

MT, medical treatment; IVT, intravenous thrombolysis; EVT, endovascular thrombectomy; SD, standard deviation; IQR, interquartile range; ASPECTS, Alberta Stroke Program Early CT Score; NIHSS, National Institutes of Health Stroke Scale; CHD, coronary heart disease; LAA, large artery atherosclerosis; CE, cardioembolism.

Following the application of stabilized IPTW to balance these baseline differences, the effective sample sizes (ESS) for the MT, IVT, and EVT groups were 87.7, 18.1, and 25.1, respectively. The detailed covariate balance before and after weighting is presented in Supplementary Table 1. Prior to weighting, several baseline characteristics exhibited significant imbalance, notably the initial NIHSS score (SMD = 0.884) and atrial fibrillation (SMD = 0.746). After IPTW, the SMDs for most covariates decreased substantially, indicating improved balance across the groups (e.g., initial NIHSS SMD decreased to 0.145). Although residual imbalance remained for variables, key clinical predictors (including age, sex, initial NIHSS score, onset-to-arrival time, ASPECTS, stroke mechanism, and successful reperfusion) were further adjusted for in the subsequent multivariable outcome models.

### Primary outcome: return to prestroke functional status

**Figure 2:**
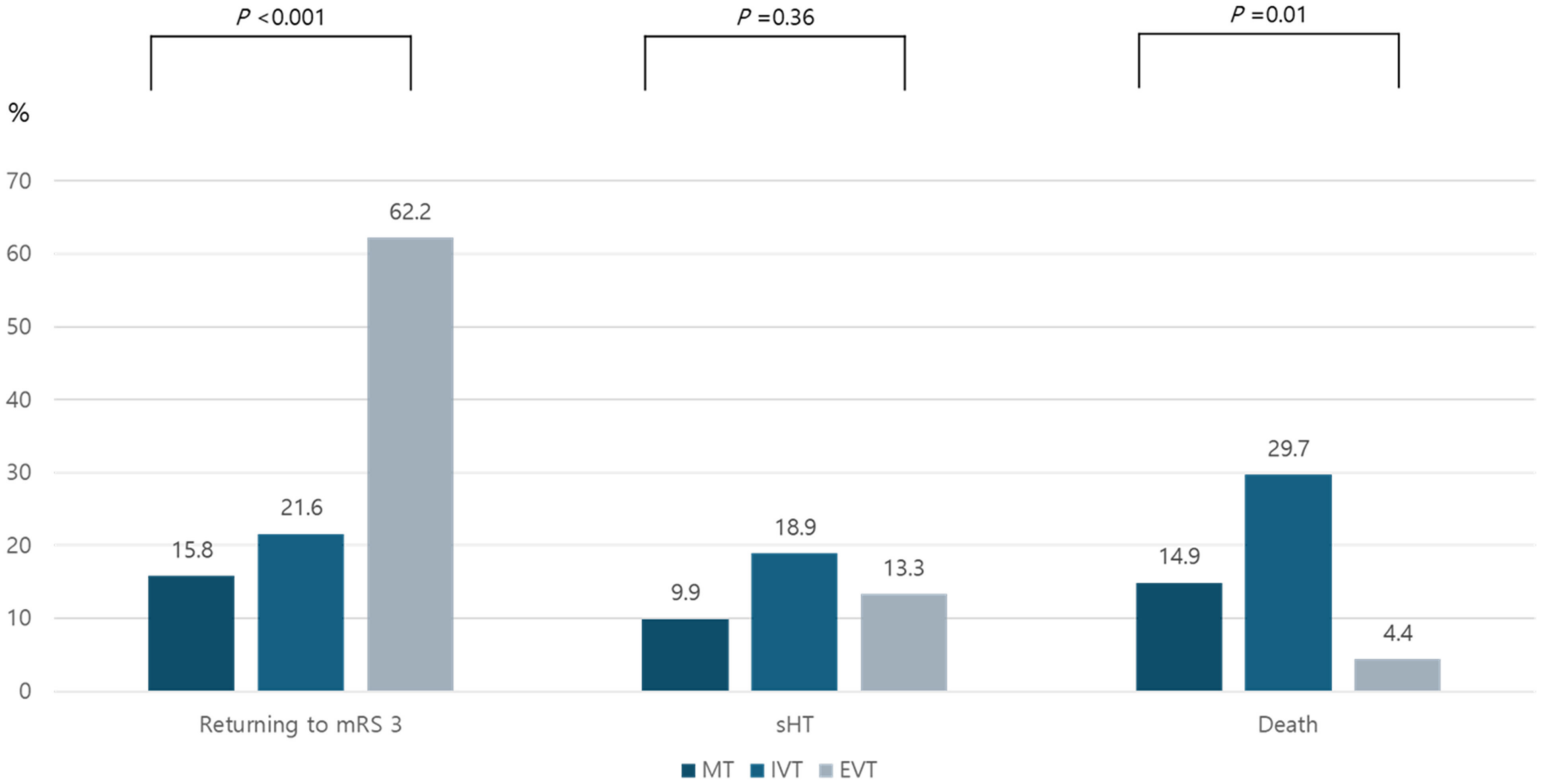
Functional outcomes and safety according to treatment strategy. mRS, modified Rankin Scale; sHT, symptomatic hemorrhagic transformation; MT, medical treatment; IVT, intravenous thrombolysis; EVT, endovascular thrombectomy.

Three months after the stroke, 15.8% of patients in the MT group, 21.6% of patients in the IVT group, and 62.2% of patients in the EVT group returned to their pre-stroke functional status (mRS = 3) (*p* < 0.001, Figure 2). IPTW-weighted multivariable logistic regression analyses, which adjusted for age, sex, initial NIHSS score, onset-to-arrival time, ASPECTS, stroke mechanism, and successful reperfusion, revealed that EVT was strongly associated with a higher likelihood of achieving the primary outcome compared with MT (adjusted odds ratio [OR] 7.63, 95% confidence interval [CI] [3.02–19.28]; *p* < 0.001). In contrast, IVT alone was not associated with functional recovery (OR 1.15, 95% CI [0.34–3.85]; *p* = 0.82) (Table 2, Supplementary Table 2). A higher initial NIHSS score was independently associated with a lower probability of a favorable outcome (OR: 0.85 per point increase; 95% CI: [0.78–0.92]; *p* < 0.001), whereas successful reperfusion was a strong positive predictor (OR: 7.83; 95% CI: [2.53–24.28]; *p* < 0.001).

**Table 2:** Multivariate analysis showing impact of EVT on outcomes according to other treatment (IPTW weighted).

Treatment	Primary outcome			Ordinal mRS shift			Death			sHT		
	OR	95% CI	*p*-value	OR	95% CI	*p*-value	OR	95% CI	*p*-value	OR	95% CI	*p*-value
MT	Reference			Reference			Reference			Reference		
IVT	1.15	0.34 – 3.85	0.82	1.44	0.63 – 3.29	0.38	2.17	0.68–6.91	0.19	1.63	0.44–5.98	0.46
EVT	7.63	3.02 – 19.28	< 0.001	0.32	0.14 – 0.70	0.004	0.45	0.10–1.95	0.29	2.11	0.69–6.44	0.19

EVT, endovascular thrombectomy; IPTW, inverse probability of treatment weighting; mRS, modified Rankin Scale; sHT, symptomatic hemorrhagic transformation; MT, medical treatment; IVT, intravenous thrombolysis.

### Ordinal mRS shift analysis

In IPTW-weighted ordinal logistic regression models, EVT was associated with a significant shift toward better functional outcomes across the full distribution of 3-month mRS scores compared to MT (common OR 0.32, 95% CI [0.14–0.70]; *p* = 0.004) (Table 2, Supplementary Table 2). IVT did not demonstrate a significant beneficial shift (common OR 1.44, 95% CI [0.63–3.29]; *p* = 0.38). As in the primary outcome analysis, a higher NIHSS score was strongly associated with worse functional outcomes (common OR: 1.16 per point increase; 95% CI: 1.11–1.22; *p* < 0.001), while successful reperfusion was associated with substantial improvement in functional outcomes (common OR: 0.25; 95% CI: 0.11–0.56; *p* < 0.001).

### Secondary outcomes

sHT occurred in 9.9% of patients in the MT group, 18.9% in the IVT group, and 13.3% in the EVT group (*p* = 0.36, Figure 2). After IPTW adjustment, neither EVT nor IVT was associated with a significantly increased risk of sHT (Table 2, Supplementary Table 2). At 3 months, mortality occurred in 14.9% of patients in the MT group, 29.7% in the IVT group, and 4.4% in the EVT group (*p* = 0.01, Figure 2). However, in IPTW-weighted analyses, neither IVT nor EVT was independently associated with mortality compared to medical treatment (EVT: OR 0.45, 95% CI [0.10–1.95]; *p* = 0.29; IVT: OR 2.17, 95% CI [0.68–6.91]; *p* = 0.19) (Table 2, Supplementary Table 3).

### EVT vs. IVT comparison

A pairwise IPTW-weighted comparison (Table 3) revealed that EVT was associated with a significantly higher likelihood of returning to pre-stroke functional status (OR: 5.17; 95% CI: 1.55–17.19; *p* = 0.01) and a significant shift toward better functional outcomes (common OR: 0.25; 95% CI: 0.08–0.73; *p* = 0.01). Successful reperfusion remained a strong independent predictor of favorable outcomes in both analyses.

**Table 3:** Multivariate analysis showing impact of EVT on 3-month mRS compared to IVT.

Variables	Primary outcome (IPTW-weighted)			Ordinal mRS shift (IPTW-weighted)		
	OR	95% CI	*p*-value	OR	95% CI	*p*-value
IVT	Reference			Reference		
EVT	5.17	1.55–17.19	0.01	0.25	0.08–0.73	0.01
Age	1.01	0.96–1.06	0.79	0.99	0.95–1.04	0.77
Male	0.87	0.23–3.29	0.84	1.11	0.37–3.35	0.85
NIHSS score	0.87	0.78–0.98	0.02	1.20	1.10–1.31	0.001
Onset-to-arrival time	1.22	0.93–1.60	0.14	0.78	0.58–1.05	0.10
ASPECTS	0.96	0.40–2.30	0.93	0.86	0.37–1.99	0.73
Successful reperfusion	8.03	1.98–32.50	0.004	0.19	0.06–0.58	0.004
Stroke mechanism						
LAA	Reference			Reference		
CE	1.14	0.27–4.81	0.86	0.43	0.14–1.31	0.14
Others	0.34	0.07–1.66	0.18	1.01	0.31–3.25	0.99

EVT, endovascular thrombectomy; mRS, modified Rankin Scale; IVT, intravenous thrombolysis; IPTW, inverse probability of treatment weighting; sHT, symptomatic hemorrhagic transformation; MT, medical treatment; ASPECTS, Alberta Stroke Program Early CT Score; NIHSS, National Institutes of Health Stroke Scale; LAA, large artery atherosclerosis; CE, cardioembolism.

## Discussion

In this study, more than half of the patients with pre-stroke mRS 3 and LVO (55.2%, or 101 out of 183 patients) did not receive reperfusion therapy, even though they arrived at the hospital within the “golden time.” Nevertheless, we found that EVT was consistently associated with a substantially higher likelihood of returning to pre-stroke functional status at 3 months. This benefit was observed in conventional multivariable-adjusted analyses and persisted after rigorous adjustment using inverse probability of treatment weighting. This suggests that the observed association is unlikely to be solely explained by treatment-selection bias. In contrast, IVT alone was not associated with functional recovery in this population. Importantly, EVT was also associated with a favorable shift across the entire distribution of three-month mRS scores, which reinforces the robustness of its functional benefit beyond a dichotomous outcome definition.

Unlike prior studies that grouped a wide spectrum of pre-stroke disability (mRS 3–5) or relied on absolute functional independence as the primary outcome, our study focuses specifically on patients with pre-stroke mRS 3. Our study adopts return to baseline function as a patient-centered endpoint, enabling a more clinically meaningful evaluation of the benefit of EVT in this population (14, 18, 19). Another strength of our study is that we compared MT, IVT, and EVT simultaneously within a single cohort of patients with a pre-stroke mRS score of 3 and confirmed LVO. Most prior studies have focused on pairwise comparisons, such as EVT vs. no EVT or EVT vs. IVT. This limits the ability to contextualize the relative effectiveness of each treatment strategy in real-world clinical decision-making. By evaluating all three therapeutic approaches together using multinomial propensity score weighting, our study provides a more comprehensive, clinically relevant framework for selecting treatments in this understudied population. The consistent functional benefit of EVT observed across multiple analytic approaches suggests that EVT can prevent further functional decline in carefully selected patients with a pre-stroke mRS score of 3 and LVO. These findings support a more individualized treatment strategy, indicating that prestroke disability alone should not automatically preclude consideration of EVT when imaging and clinical features suggest a reasonable likelihood of maintaining baseline functional status.

Overall, the rates of sHT were higher in the IVT and EVT groups than in the MT group. However, after IPTW adjustment, neither EVT nor IVT was independently associated with an increased risk of sHT. This finding is noteworthy given the higher baseline stroke severity among patients receiving reperfusion therapy. It suggests that hemorrhagic risk may be driven more by baseline tissue vulnerability than by the treatment modality itself. Previous studies have consistently demonstrated that factors such as infarct burden and blood-brain barrier integrity play a pivotal role in hemorrhagic transformation development (20, 21). In line with this concept, a higher ASPECTS score was independently associated with a lower risk of sHT in our study. This finding indicates that a preserved ischemic core and viable tissue mitigate hemorrhagic complications (22, 23). The relatively higher overall rate of sHT observed in our cohort compared to prior studies should be interpreted in the context of our population's distinct clinical profile. All patients had a pre-stroke mRS score of 3 and confirmed LVO, a combination associated with greater baseline vulnerability, including higher stroke severity and reduced neurological reserve. Additionally, patients with pre-stroke disability tend to be older and have a higher burden of comorbidities, which may predispose them to blood-brain barrier disruption following ischemia-reperfusion. Methodological factors may also have contributed to the higher rate of sHT observed in our study, as we employed systematic imaging follow-up and rigorous sHT adjudication, which may have increased detection compared with studies using less stringent definitions. Importantly, successful reperfusion itself was not independently associated with sHT. This finding supports the notion that reperfusion injury is not inevitable when appropriate imaging-based patient selection limits treatment to those with salvageable brain tissue (24, 25). In our study, we selected patients for EVT mainly because they arrived early (within 4.5 h) and had a large vessel occlusion confirmed by CTA. Some might argue that vulnerable patients with a pre-stroke mRS of 3 need advanced imaging (such as CT perfusion or MRI) to safely decide on EVT. However, a recent multicenter study demonstrated that even for large-core strokes—another difficult patient group—standard noncontract computed tomography (NCCT) is enough, and patient outcomes were just as good as those with advanced imaging (26). These findings suggest that in the early window, standard NCCT is enough to safely justify EVT, even for those with moderate disability.

Regarding mortality, stroke severity at the time of admission, as reflected by the NIHSS score, was the strongest predictor of death across all analyses. Treatment strategy, however, was not (27). Despite their greater baseline severity, the absence of excess mortality in EVT-treated patients suggests that timely restoration of cerebral perfusion may counterbalance the otherwise poor natural history of LVO (28). Patients with a pre-stroke mRS score of 3 may have enough physiological reserve to withstand reperfusion-related stress without experiencing significant hemorrhagic or fatal complications. This distinguishes them from patients with more severe pre-stroke disability (29, 30).

Notably, IVT was not associated with meaningful functional benefits compared to EVT. This may be partly explained by IVT's lower rate of successful reperfusion in patients with LVO (31). Consistent with prior literature, successful recanalization occurred less frequently after IVT than after EVT (46.9 vs. 60%), reflecting the inherent limitations of pharmacological thrombolysis in achieving timely and durable reopening of proximal occlusions. Previous observational studies have reported similar findings: attenuated or inconsistent functional benefits of IVT in patients with prestroke disability, particularly when LVO is present. In contrast, EVT has been more consistently associated with preservation of baseline function when outcomes are defined relative to prestroke status (13, 32, 33). For patients with a pre-stroke mRS score of 3, the therapeutic goal is to prevent functional decline rather than achieve full recovery. In these patients, incomplete or delayed reperfusion following IVT may be insufficient to prevent irreversible injury to functionally critical brain regions. Furthermore, the clinical relevance of reperfusion differs substantially between treatment modalities. EVT aims to immediately and definitively restore flow at the site of occlusion. However, reperfusion after IVT is susceptible to residual thrombus, distal embolization, and early re-occlusion. These factors can limit effective microvascular reperfusion, even when recanalization is achieved (34). Our finding that successful reperfusion, rather than IVT exposure, was the dominant predictor of functional recovery supports this mechanistic distinction (35). Although our sample size was relatively modest, which limited the statistical power of certain subgroup and safety analyses, the consistency of the observed functional benefits of EVT across multiple analytic approaches strengthens the clinical relevance of our findings. Together, these results imply that EVT may be more effective than IVT in preserving baseline functional status in patients with prestroke mRS 3 and confirmed LVO by more reliably addressing the underlying pathophysiological substrate of ischemia.

There are several limitations to this study that should be acknowledged. First, it was a retrospective observational analysis. Although inverse probability of treatment weighting was applied to mitigate confounding by indication, our propensity score model lacked certain key determinants of treatment allocation relevant to patients with prestroke disability, such as frailty, baseline cognitive status, and living situations. Because these variables are not captured in the registry, residual confounding by indication from these unmeasured variables cannot be ruled out. Second, treatment allocation was not randomized and may have been influenced by factors such as clinician judgment, perceived frailty, or subtle imaging features not fully captured in the registry. Third, the sample size was relatively small, especially for subgroup and safety analyses. This may have limited the study's statistical power to detect small differences in outcomes, such as symptomatic hemorrhagic transformation or mortality. And, the EVT cohort combined direct EVT with bridging IVT, and anterior with posterior circulation strokes. Although this reflects a standard endovascular strategy, the limited number of cases precluded meaningful subgroup analyses to address this intra-group heterogeneity. So, nonsignificant findings for secondary outcomes should be interpreted with caution. Fourth, the assessment of successful reperfusion differed by treatment modality. Reperfusion after IVT was evaluated using follow-up CTA or MRA, whereas reperfusion after EVT was assessed immediately by procedural angiography. This difference in timing and methodology may have introduced measurement heterogeneity and affected the comparability of reperfusion metrics across treatment groups. Furthermore, while variables such as procedural time and reperfusion status are key determinants of EVT and IVT outcomes, they could not be integrated into our primary comparative analyses. Because these metrics are modality-specific and not applicable across all treatment arms (e.g., absent in the MT group), incorporating them into a unified between-group comparison was not feasible. Therefore, the specific impact of these procedural factors on the overall outcomes could not be directly evaluated. Finally, this study focused exclusively on a highly specific clinical subgroup: patients with a prestroke mRS score of 3 and confirmed large vessel occlusion. The substantial reduction from the initially screened population to our final analytic cohort was primarily driven by these strict eligibility criteria, which were necessary to directly address our core clinical question. However, we acknowledge that this rigorous targeting resulted in a highly selected cohort, which inherently limits the external validity of our findings. This focus may limit the generalizability of our findings to broader, unselected real-world populations, as well as to patients with more severe prestroke disability or to those with medium vessle occlusion. Prospective studies with larger sample sizes and standardized reperfusion assessments are needed to validate and extend our findings.

In patients with acute ischemic stroke due to large vessel occlusion (LVO) and moderate pre-stroke disability (mRS 3), EVT was associated with a substantially higher likelihood of returning to pre-stroke functional status and a favorable shift in functional outcomes compared with MT or IVT alone. These benefits were observed without an increased risk of stroke or mortality after rigorous adjustment for baseline differences. Our findings suggest that timely and functionally relevant reperfusion, beyond recanalization alone, is critical for preserving baseline function. This supports the consideration of EVT in carefully selected patients with moderate pre-stroke disability.

## Data Availability

The datasets generated and/or analysed during the current study are available from the corresponding author on reasonable request.
