# Supplementary material for: Endovascular thrombectomy in patients with acute ischemic stroke and prestroke mRS 3: a multicenter IPTW analysis focused on functional preservation
Source: Front Neurol. 2026 May 22;17:1841144. doi: 10.3389/fneur.2026.1841144 (PMC13236520; doi:10.3389/fneur.2026.1841144)
Supplement: Supplementary file 1 [file Table_1.docx]

Supplementary table 1. Maximum standardized mean differences (SMD) of baseline characteristics before and after inverse probability of treatment weighting (IPTW)

| **Characteristic** | **Before IPTW Max SMD** | **After IPTW Max SMD** |
| --- | --- | --- |
| Age | 0.316 | 0.360 |
| Male | 0.460 | 0.183 |
| Initial NIHSS | 0.884 | 0.145 |
| Onset-to-arrival time | 0.646 | 0.481 |
| ASPECTS | 0.260 | 0.191 |
| History of stroke | 0.411 | 0.172 |
| History of CHD | 0.295 | 0.196 |
| Hypertension | 0.397 | 0.248 |
| Diabetes mellitus | 0.152 | 0.165 |
| Atrial fibrillation | 0.746 | 0.128 |
| Hyperlipidemia | 0.197 | 0.215 |
| Smoking | 0.611 | 0.544 |
| Prior antiplatelet | 0.334 | 0.094 |
| Prior anticoagulation | 0.355 | 0.010 |
| Prior statin | 0.336 | 0.346 |
| Stroke mechanism | 0.319 | 0.094 |

Supplementary table 2. Multivariate analysis showing impact of EVT on 3-month mRS according to other treatment.

|  | Primary outcome (IPTW-weighted) | | | Ordinal mRS shift (IPTW-weighted) | | |
| --- | --- | --- | --- | --- | --- | --- |
|  | OR | 95% CI | p-value | Common OR | 95% CI | p-value |
| MT | Reference | | | Reference | | |
| IVT | 1.15 | 0.34 – 3.85 | 0.818 | 1.44 | 0.63 – 3.29 | 0.382 |
| EVT | 7.63 | 3.02 – 19.28 | <0.001 | 0.32 | 0.14 – 0.70 | 0.004 |
| Age | 0.98 | 0.95 – 1.02 | 0.358 | 1.01 | 0.98 – 1.03 | 0.617 |
| Male | 0.37 | 0.15 – 0.93 | 0.034 | 1.59 | 0.86 – 2.92 | 0.139 |
| NIHSS score | 0.85 | 0.78 – 0.92 | <0.001 | 1.16 | 1.11 – 1.22 | <0.001 |
| Onset-to-arrival | 1.1 | 0.88 – 1.39 | 0.401 | 0.85 | 0.71 – 1.02 | 0.073 |
| ASPECTS | 1.36 | 0.72 – 2.60 | 0.345 | 0.78 | 0.49 – 1.23 | 0.284 |
| Successful reperfusion | 7.83 | 2.53 – 24.28 | <0.001 | 0.25 | 0.11 – 0.56 | <0.001 |
| Stroke mechanism |  |  |  |  |  |  |
| LAA | reference | | | Reference | | |
| CE | 1.42 | 0.56 – 3.64 | 0.463 | 0.76 | 0.39 – 1.49 | 0.421 |
| Others | 0.59 | 0.19 – 1.86 | 0.369 | 0.84 | 0.41 – 1.73 | 0.636 |

Supplementary table 3. Multivariate analysis showing impact of EVT on secondary outcomes according to other treatment.

|  | Death (IPTW-weighted) | | | sHT (IPTW-weighted) | | |
| --- | --- | --- | --- | --- | --- | --- |
|  | OR | 95% CI | p-value | OR | 95% CI | p-value |
| MT | Reference | | | Reference | | |
| IVT | 2.17 | 0.68–6.91 | 0.189 | 1.63 | 0.44–5.98 | 0.463 |
| EVT | 0.45 | 0.10–1.95 | 0.286 | 2.11 | 0.69–6.44 | 0.192 |
| Age | 1 | 0.96–1.04 | 0.896 | 1.00 | 0.96–1.04 | 0.911 |
| Male | 0.9 | 0.31–2.61 | 0.842 | 3.73 | 1.22–11.37 | 0.021 |
| NIHSS score | 1.11 | 1.03–1.19 | 0.0052 | 0.98 | 0.91–1.06 | 0.666 |
| Onset-to-arrival | 1 | 0.77–1.30 | 0.992 | 1.07 | 0.84–1.36 | 0.61 |
| ASPECTS | 0.65 | 0.30–1.39 | 0.266 | 0.5 | 0.25–0.99 | 0.048 |
| Successful reperfusion | 0.28 | 0.08–1.02 | 0.053 | 1.26 | 0.37–4.26 | 0.715 |
| Stroke mechanism |  |  |  |  |  |  |
| LAA | Reference | | | Reference | | |
| CE | 0.78 | 0.27–2.25 | 0.64 | 0.62 | 0.20–1.97 | 0.423 |
| Others | 0.46 | 0.11–1.84 | 0.27 | 0.69 | 0.18–2.68 | 0.597 |
